# Cryptic Diversity and Venom Glands in Western Atlantic Clingfishes of the Genus *Acyrtus* (Teleostei: Gobiesocidae)

**DOI:** 10.1371/journal.pone.0097664

**Published:** 2014-05-13

**Authors:** Kevin W. Conway, Carole Baldwin, Macaulay D. White

**Affiliations:** 1 Department of Wildlife and Fisheries Sciences and Biodiversity Research and Teaching Collections, Texas A&M University, College Station, Texas, United States of America; 2 Department of Vertebrate Zoology, National Museum of Natural History, Smithsonian Institution, Washington D. C., United States of America; 3 Undergraduate Degree Program, Department of Wildlife and Fisheries Sciences, Texas A&M University, College Station, Texas, United States of America; Instituto Butantan, Brazil

## Abstract

Examination of genetic data (mitochondrial cytochrome c oxidase I) for western Atlantic clingfishes revealed two distinct lineages within a group of individuals originally identified as *Acyrtus artius.* Subsequent investigation of preserved voucher specimens was conducted to reconcile the genetic data and the existing classification, which is based on morphology. In addition to discovering that one of the genetic lineages is an undescribed species, which we describe as *Acyrtus lanthanum,* new species, we found that the nominal species *Acyrtus artius* has a putative venom gland associated with the subopercle that has been overlooked since the species was described nearly 60 years ago. The new species lacks the subopercular gland as does *Acyrtus rubiginosus*, but one is present in the related *Arcos nudus*. Venom glands have not been reported previously for the Gobiesocidae, and the venom gland described herein for *Acyrtus* and *Arcos* represents the first example in teleost fishes of a venom gland associated with the subopercle.

## Introduction

Small bodied, cryptobenthic marine fishes (<50 mm in length and closely associated with the benthos [1]) represent a diverse and often overlooked component of global marine vertebrate biodiversity. Due to the difficulties that are often associated with the collection [1]–[6] and identification [7]–[8] of cryptobenthic species, it is not surprising that the majority of newly described marine vertebrates falls within this category. Even in relatively well studied regions of the world’s oceans, new species of cryptobenthic fishes are discovered on an annual basis [7]–[10], and an increasing number of DNA-based studies are revealing that even relatively well-known species of cryptobenthic fishes represent complexes of morphologically similar, cryptic species [11]–[15].

*Acyrtus* Schultz [16] is one of several New World genera of the Gobiesocidae (clingfishes) composed solely of tiny, cryptobenthic species, attaining maximum sizes of less than 30 mm [17]–[19]. Two of the three currently described species of *Acyrtus*, *A. rubiginosus* (Poey) and *A. artius* Briggs, are widely distributed throughout the Bahamas and Caribbean and have been relatively well studied compared to other western Atlantic clingfishes [20]–[22]. Preliminary analyses of sequence data from the mitochondrial cytochrome c oxidase I (COI) gene obtained from individuals identified as *Acyrtus artius* from the coast of Belize revealed unexpectedly high levels of genetic diversity between individuals collected from shallow (<5 m) lagoon areas and coral rubble zones and those from deeper (8–20 m) spur and groove areas and on walls of outer ridges. Further investigation of this material and additional specimens identified as *Acyrtus artius* from throughout the Caribbean and Bahamas revealed a number of morphological differences between specimens collected from shallower versus deeper depths, including differences in head and disc morphology. Most notably, specimens from deeper depths exhibit a large, opaque patch of skin along the medial face of a spine-like process on the subopercle. This last feature is absent from individuals identified as *Acyrtus artius* from shallower water but is present in the type material of *Acyrtus artius.* Based on these differences we describe the form inhabiting shallow waters as a new species of *Acyrtus* and redescribe *A. artius*, which we hypothesize is a putatively venomous member of the Gobiesocidae.

## Methods

### Morphological Investigation

**Figure 1:**
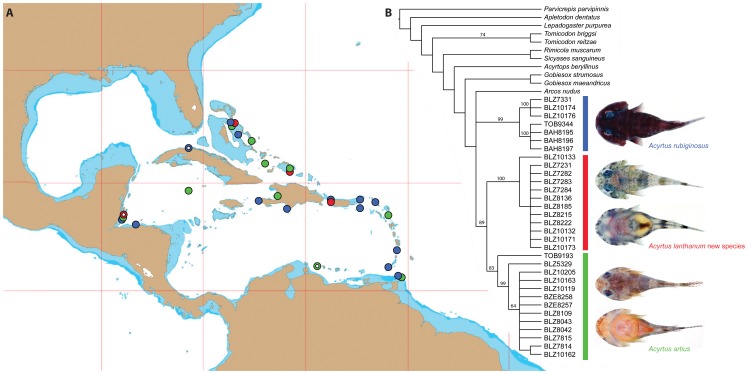
A, Collection localities of *Acyrtus* material examined as part of this investigation. Color of symbols correspond to clades in B. Open symbols represent type localities. B, Strict consensus of 479 equally parsimonious cladograms (1102 steps; CI = 0.391; RI = 0.681) resulting from parsimony analysis of COI data set. Numbers above branches represent bootstrap support values.

Specimens of *Acyrtus* utilized in this study (Fig. 1A) were obtained during recent fieldwork throughout the western Central Atlantic, including Belize, Bahamas, Tobago (Trinidad and Tobago), and Turks and Caicos, as part of an ongoing investigation of Caribbean reef fish diversity [11]–[12], [23]–[24]. Additional specimens were also obtained from museum collections [25] (listed in Information S1).

Measurements obtained from specimens generally follow those of Briggs [17], with the addition of predorsal, preanal and preanus lengths (which are the shortest distances between the tip of the upper lip and the dorsal-fin origin, anal-fin origin, and anus, respectively). As reported previously [26], we found relatively few of the measurements reported by Briggs to be useful in distinguishing between the species we investigated, and we report here only on the following: (1) standard length (SL), (2) head length (HL), (3) body depth (taken at dorsal-fin origin), (4) predorsal length, (5) preanal length, (6) preanus length, (7) distance between anus and posterior margin of disc, (8) distance between anus and anal-fin origin, (9, 10) caudal-peduncle length and depth, (11, 12) disc length and width, (13) head depth through orbit, (14, 15) head width through orbit and through widest part of head, (16) interorbital width, (17) snout length, and (18) eye diameter. Measurements are expressed as a percentage of either SL (measurements 2–12) or HL (measurements 13–18).

Selected specimens were cleared and double stained (c&s) for bone and cartilage investigation [27]. Counts were obtained only from cleared and stained specimens and generally follow those of Williams and Tyler [26] with the following exceptions: we use the term “abdominal” (vs. “precaudal”) to refer to those vertebrae situated anterior to caudal vertebrae and “epicentral” (vs. “epineural”) for the single series of intermuscular bones present along the horizontal septum [28]. Numbers of incisors and canines in the upper and lower jaws are reported separately (with incisors reported separately for the left and right sides of each jaw using the formula left+right). Numbers provided in parentheses after a particular count represent the number of cleared and stained specimens that exhibit that count. Caudal-fin rays are identified as principal or procurrent following previous authors [29]–[30]. Adhesive disc and cephalic sensory pore terminology follows Briggs [17] and Shiogaki and Dotsu [31], respectively. Photographs of specimens or parts thereof were obtained using a ZEISS SteREO Discovery V20 stereomicroscope equipped with a ZEISS Axiocam MRc5 digital camera.

The opercular apparatus of the right side of selected individuals was removed and prepared for histological examination after decalcification [32]. Prepared slides were stained with hematoxylin and eosin and then examined and photographed using a ZEISS Primo Star compound microscope equipped with an Axiocam ERc5s digital camera.

### Molecular Laboratory Work and Analysis of Sequence Data

Protocols for DNA extraction and subsequent amplification of the cytochrome oxidase 1 (COI) gene from tissue samples follow those utilized by recent studies on Caribbean fishes [11]–[12], [23]–[24]. In addition to members of the genus *Acyrtus*, we also obtained COI data for eight other New World gobiesocid taxa included within the subfamily Gobiesocinae (*Acyrtops beryllinus*, *Arcos nudus*, *Gobiesox maeandricus*, *G. strumosus*, *Rimicola muscarum*, *Sicyases sanguineus, Tomicodon briggsi* and *T. reitzae*), two from the Lepadogastrinae (*Apletodon dentatus* and *Lepadogaster purpurea*), and one from the Diplocrepinae (*Parvicrepis parvipinnis*), which served as outgroups in phylogenetic analyses. Obtained sequences were aligned by eye using TextWrangler vs. 2.3 (Barebones Software Inc). The aligned data set was subsequently viewed in MacClade vs. 4.05 [33] to check for spurious stop codons and trimmed to ensure all taxa had sequences of similar length, resulting in a final aligned data set 621bp in length. GenSeq nomenclature for DNA sequences [34] and GenBank information are presented along with museum catalog numbers for voucher specimens in Table S1.

Maximum parsimony (MP) analysis of the final data set was conducted with heuristic searches in PAUP* v. 4.0b10 [35], utilizing tree-bisection and reconnection branch swapping (TBR) with the MULTREES option effective for 1000 random addition sequence replicates. All characters were equally weighted and left unordered. The resulting equally parsimonious cladograms were rooted using *P. parvipinnis* and summarized using a strict consensus method. Nodal support was estimated using non-parametric bootstrapping [36] for 1000 pseudoreplicates, utilizing a random addition sequence and TBR branch swapping.

Means of the corrected genetic distances within and between genetic lineages of *Acyrtus* corresponding to exclusive lineages obtained in resulting phylogenetic hypotheses were calculated with MEGA4.0 [37], using “within group means”, “between groups means”, and “net between groups means” options. Standard errors of the genetic distances were calculated using 1000 bootstrap replicates.

### Nomenclatural Acts

The electronic edition of this article conforms to the requirements of the amended International Code of Zoological Nomenclature, and hence the new name contained herein is available under that Code from the electronic edition of this article. This published work and the nomenclatural acts it contains have been registered in ZooBank, the online registration system for the ICZN. The ZooBank LSIDs (Life Science Identifiers) can be resolved and the associated information viewed through any standard web browser by appending the LSID to the prefix “http://zoobank.org/”. The LSID for this publication is: urn:lsid:zoobank.org:pub:0A14FC62-63B8-4758-A375-44FAD09405BD. The electronic edition of this work was published in a journal with an ISSN, and has been archived and is available from the following digital repositories: PubMed Central and LOCKSS.

### Ethics Statement

This study was carried out under Smithsonian Animal Care and Use Committee (ACUC) approval to C. C. Baldwin (ACUC #2011-07). Guidelines for field activities with wild fishes established by the American Society of Ichthyologists and Herpetologists (http://www.asih.org/sites/default/files/documents/Resources/guidelinesfishresearch2003-draft.pdf) were followed for all field collecting activities, including euthanasia with tricaine methane sulfate (MS-222). The field studies involved no endangered or protected species.

## Results

### Analyses of COI Sequence Data

Parsimony analysis of the COI data set recovered 479 equally parsimonious cladograms, each 1102 steps with consistency and retention indices of 0.391 and 0.681 respectively. Of the 621 characters included in our COI data set, 228 were identified as parsimony-informative, 361 were identified as constant, and 32 variable characters were identified as parsimony-uninformative. The strict consensus tree resulting from the 479 equally parsimonious cladograms is shown in Figure 1B. Though *Acyrtus* was not recovered as a monophyletic group in the resulting strict consensus tree (due to the placement of *Arcos nudus*), two groups of *Acyrtus* were consistently present in each of the resulting equally parsimonious cladograms and were also present in the 50% majority rule cladogram summarizing the results of the bootstrap analysis. One of these groups represents *Acyrtus rubiginosus*, and the other is composed of specimens originally identified as *Acyrtus artius*. The latter are further divided into two groups, including those collected from shallow (<5 m) lagoon areas and coral rubble zones or from deeper (8–20 m) spur and groove areas and on walls of outer ridges. The mean genetic distance between the two groups of specimens is 8.4%, and the mean, within-group genetic distances ranges from 0.5–1.3% (Table 1). The value of 8.4% is consistent with species-level variation in COI for many fish species that have been investigated previously. For example, the average intrageneric variation in COI for 207 species of Australian fishes is 9.93% [38] and 8.30% for 193 species of Canadian freshwater fishes [39].

**Table 1:** Means of corrected genetic distances within and between three genetic lineages of *Acyrtus* based on COI sequence data.

*Acyrtus* spp.	*artius* (N = 10)	*lanthanum*, n. sp. (N = 10)	*rubiginosus* (N = 7)
*Artius*	**1.3%**		
*lanthanum*, n. sp.	8.4%	**0.5%**	
*rubiginosus*	13.8%	12.6%	**3.5%**

Within-group values are in bold.

### Taxonomy

The two genetic lineages of *Acyrtus* are further differentiated by a number of morphological (see below; Table 2) and ecological (depth of capture) differences, and we consider them as distinct species. Based on our examination of the type material of *Acyrtus artius*, we refer the specimens collected from greater depths to this species and those from shallower depths to a new species, both of which are described below.

**Table 2:** Summary of differences among the four species of *Acyrtus.*

Character	*Acyrtus artius*	*Acrytus lanthanum,* n. sp.	*Acyrtus pauciradiatus*	*Acyrtus rubiginosus*
Posterolateral marginof disc region C	Papillae present	Papillae present	Papillae present	Papillae absent
Anterolateral marginof disc region C	Papillae present	Papillae absent	Papillae present	Papillae absent
Disc length	29–36% SL	31–39% SL	27–34% SL	27–32% SL
Head length	42–47% SL	40–43% SL	40–43% SL	33–40% SL
Subopercular spine	Large; grooved ventrally	Small; circular incross-section	Large; grooved ventrally	Small; circularin cross-section
Skin medial to subopercle	Opaque and granular	Undifferentiated	Opaque and granular	Undifferentiated
Branchiostegal membrane	Lateral pocket between 6^th^branchiostegal rayand subopercle	Without pocket	Lateral pocket between 6^th^branchiostegal rayand subopercle	Without pocket
Pectoral-fin rays	24–27	24–25	20–22	24–27

Data for *A. pauciradiatus* taken from Sampaio et al. [15].

### Taxonomic Accounts

#### *Acyrtus lanthanum*, new species

Urn:lsid:zoobank.org:act:7C4D9F03-66FA-4A4D-8690-12F9AD174441.

Orange spotted clingfish.

### Holotype

FMNH 84325, 14.0 mm SL, Belize: Caribbean Sea, Glover’s Reef, near mouth of Middle Cay, between Middle and Long Cays, 7 m, D. W. Greenfield et al., 16 June 1978.

### Paratypes

*Belize:*(DNA vouchers) USNM 403189, DNA number BLZ 8215, 8.0 mm SL, Carrie Bow Cay, south end, 0-2 m, field number CB08-20, L. Weigt, 21 May 2008; USNM 403477, DNA number BZE 7231, 12.5 mm SL, Carrie Bow Cay lagoon, 16° 48′ 08.00″ N, 88° 04′ 54.00″ W, 0–3 m, field number CB07-22, L. Weigt, 18 January 2007; USNM 403478, DNA number BZE 8185, 10.0 mm SL, Whale Shoals, South Cut, in and out, 16° 45′ 35.00″ N, 88° 04′ 34. 00″ W, 0–5 m, field number CB08-17, C. Baldwin et al., 20 May 2008; USNM 403480, DNA number BZE 7283,19.0 mm SL, Carrie Bow Cay, south end, 16° 48′ 08.00″ N, 88° 04′ 54.00″ W, 0–2 m, field number CB07-28, C. Baldwin & L. Weigt, 19 January 2007; USNM 403490, DNA number BZE 7282, 18.0 mm SL, Carrie Bow Cay, south end, 16° 48′ 08.00″ N, 88° 04′ 54.00″ W, 0–2 m, field number CB07-28, C. Baldwin & L. Weigt, 19 January 2007; USNM 403497, DNA number BLZ 8222, 10.0 mm SL, Curlew Cay, 0–3 m, field number CB08-19, C. Baldwin & Z. Foltz, 21 May 2008; USNM 403499, DNA number BZE 8136, 18.0 mm SL, Glovers Reef, west side, 16° 43′ 08.00″ N, 87° 53′ 13.00″ W, 0–3 m, field number CB08-11, C. Baldwin et al., 18 May 2008; USNM 404132, DNA number BLZ 10132, 12.5 mm SL, Carrie Bow Cay, south side, 1.5–3 m, field number CB10-15, C. Castillo & D. Griswold, 13 November 2010; USNM 404133, DNA number BLZ 10133, 13.0 mm SL, Carrie Bow Cay, south side, 1.5–3 m, field number CB10-15, C. Castillo & D. Griswold, 13 November 2010; USNM 404171, DNA number BLZ 10171, 16.5 mm SL, Carrie Bow Cay, south side, 1–2 m, field number CB10-18, C. Castillo & D. Griswold, 14 November 2010; USNM 404172, DNA number BLZ 10172, 17.0 mm SL, Carrie Bow Cay, south side, 1–2 m, field number CB10-18, C. Castillo & D. Griswold, 14 November 2010; USNM 404173, DNA number 10173, 15.0 mm SL, Carrie Bow Cay, south side, 1–2 m, field number CB10-18, C. Castillo & D. Griswold, 14 November 2010; (non-DNA vouchers) FMNH 124190, 2, 8.8–13.3 mm SL, same data as holotype; FMNH 84334, 6, 14.5–16.9 SL, Carrie Bow Cay, Barrier Reef, D.W.Greenfield & T.A. Greenfield, 11 May 1977.

### Additional Material

*Bahamas:* ANSP 81310, 3, 19.5–23.8 mm SL, Great Bahama Bank, Treasure Island (Salt Cay), South shore of west tip, J.E. Böhlke et al., 14 August 1955; ANSP 115017, 4, 11.0–20.7 mm SL, Great Bahama Bank, Sandy Cay, 25° 7′ 0.00″ N, 77° 13′ 0.00″ W, J.E. Böhlke et al., 11 August 1969; UF 212697, 4 (2 c&s), 12.6–20.9 mm SL, Exuma Sound, East side of small cay northwest of Little Majors Spot Cay, H. Feddern et al., 25 August 1963. *Puerto Rico (US)*: ANSP 144517, 5, 13.2–18.9 mm SL, Isla Desecheo, small bay at SW side, J.E. Randall, 6 March 1965; ANSP 144518, 1, 12.9 mm SL, Isla Desecheo, north side of island, J.E. Randall, 6 March 1965. *Turks and Caicos*: USNM 403501, 14.0 mm SL, South Caicos, East Bay, 21° 32′ 15.00″ N, 71° 28′ 48.00″ W, 0–5 m, field number TCI 09–09, J. Williams et al., 9 October 2009; USNM 403504, 16.0 mm SL, South Caicos, East Bay, 21° 32′ 15.00″ N, 71° 28′ 48.00″ W, 0–5 m, field number TCI 09-09, J. Williams et al., 9 October 2009.

### Diagnosis

A member of the genus *Acyrtus* distinguished from *A. artius* and *A. pauciradiatus* by having the branchiostegal membrane continuous with the operculum (vs. a deep pocket between the branchiostegal membrane and the operculum), skin medial to subopercle thin and undifferentiated (vs. large opaque patch of skin associated with the medial face of the subopercle, caused by a dense aggregation of large, tightly packed clavate cells in the epidermis), a poorly developed subopercular spine (vs. subopercular spine elongate, with a well-developed ventral groove), and anterolateral margin of disc region C without papillae (vs. two widely separated clusters of papillae along the anterolateral margin of disc region C). It is further distinguished from *A. pauciradiatus* by having more pectoral-fin rays (24–25 vs. 20–22) and by fresh color pattern (with variously colored saddles on the trunk vs. uniformly pale reddish pink). From *A. artius, A. lanthanum* also differs by usually having a shorter head (head length 40–43% SL vs. 44–47), a deeper head (head depth at orbit 50–60% HL vs. 38–48) and usually a broader head (head width at orbit 78–89% HL vs. 68–78%); by the shape of the ventral postcleithrum (tip of posteromedial arm rounded vs. scalloped); and by fresh color pattern–most notably having small, well-spaced, orange to red spots or dashes (vs. larger, more oblong, and denser dashes) on the dorsal and lateral portions of the trunk that do not extend ventrally to the area in front of anal-fin origin (vs. extending ventrally to area in front of anal-fin origin); lacking a bar or blotch of pigment between the saddle of pigment at the base of the caudal fin and the one beneath the anterior portion of the dorsal fin (vs. having a blotch or bar of pigment here); and in having a mostly dark (blue to black) iris with reddish-gold inner ring (vs. a mostly or entirely red iris). *Acyrtus lanthanum* usually can be distinguished from *A. rubiginosus* by its larger adhesive disc (disc length 31–39% SL vs. 27–32% SL; disc width 31–38% SL vs. 22–31% SL), and it can further be distinguished by the absence of large coniform teeth anterolaterally in upper jaw (vs. presence), fewer teeth in the lower jaw (3–4 small coniform teeth posterior to larger, anterolaterally placed coniform teeth vs. 7–8), a single row of pharyngeal teeth associated with ceratobranchial 5 (vs. 2 rows), the presence of paired clusters of papillae posteriorly in disc region C (vs. absence), presence of small round papillae along the anterior edge of disc region B (vs. large, irregular shaped papillae), by the shape of the ventral postcleithrum (anterior arm slender and uniform in diameter along entire length vs. anterior arm thicker proximally, decreasing in diameter distally), and by fresh color pattern, most notably in having a mostly white background body color (vs. purple).

### Description

**Figure 2:**
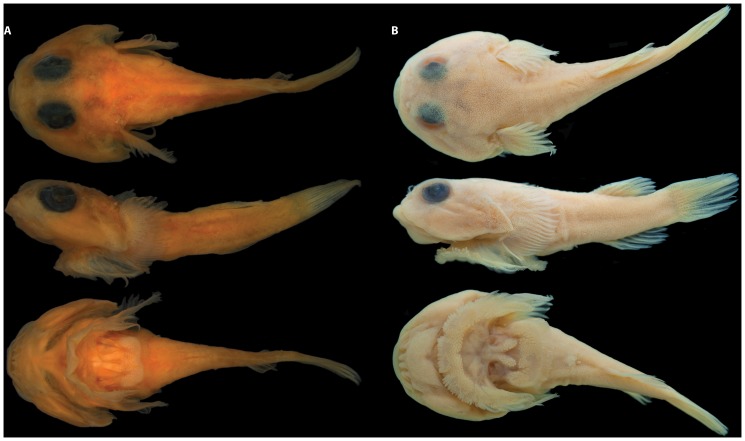
*Acyrtus lanthanum*, new species. A, holotype, FMNH 84325, 14.0

**Figure 3:**
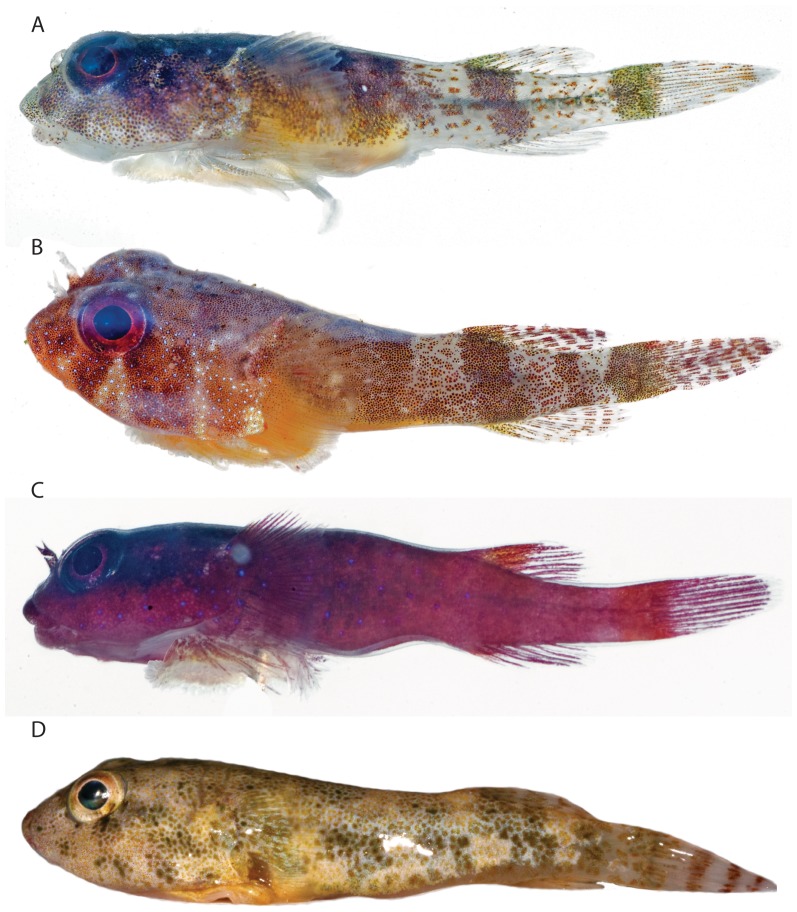
Recently collected specimens of *Acyrtus* and *Arcos*. A, *Acyrtus lanthanum*, new species, USNM 404171, 16.5 mm SL, DNA # BLZ 10171, Belize. B, *Acyrtus artius*, USNM 404205, 19.0 mm SL, DNA # BLZ 10205, Belize. C, *Acyrtus rubiginosus*, USNM 404174, 10.5 mm SL, DNA # BLZ 10174, Belize. D, *Arcos nudus,* USNM 403507, 49.7 mm SL, DNA# ELU 1003, Bahamas. Photographs A-C by Donald Griswold and Carole Baldwin; D by Louis Johnson, edited by authors.

**Figure 4:**
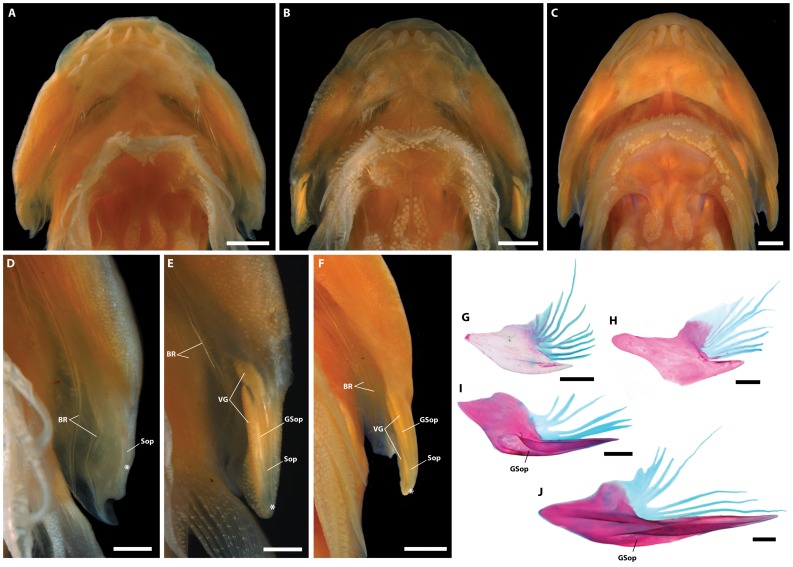
Ventral view of head (A–B), subopercular region of left side (D–F), and subopercle from right side in medial view (G–J) in members of *Acyrtus* and *Arcos*. A, *Acyrtus lanthanum*, new species, holotype, FMNH 84325, 14.0 mm SL. B, *Acyrtus artius*, ANSP 123658, 15.2 mm SL. C, *Arcos nudus*, ANSP 115602, 25.6 mm SL. D, Close up of subopercular region of left side in A. E, Close up of subopercular region of left side in B. F, Close up of subopercular region of left side in C. G, *Acyrtus lanthanum,* new species, ANSP 106336, 20.4 mm SL. H, *Acyrtus rubiginosus*, UF 149202, 20.0 mm SL. I, *Acyrtus artius*, ANSP 94757, 24.2 mm SL. J, *Arcos nudus*, ANSP 142945, 27.0 mm SL. White asterisks indicate posteriormost tip of subopercular spine in D–E. Abbreviations: BR, branchiostegal ray; GSop, groove in subopercle; Sop, subopercle; VGS, venom gland cells. Scale bars equal to 1 mm (A–C, F) or 400 µm (D–E, G–J).

General body shape as in Figures 2 and 3A. Morphometric data are listed in Table 3 and selected counts in Table 4. Head large, slightly dorsoventrally compressed. Body moderately dorsoventrally compressed anteriorly; becoming increasingly laterally compressed posteriorly. Body deepest midway between head and dorsal-fin origin. Eye large, positioned in upper half of head; center of eye closer to tip of snout than to posterior margin of operculum. Snout short. Anterior nostril tubular, with small, often bifurcated, cirri extending from posterior margin. Posterior nostril surrounded by a low fleshy rim; situated close to base of anterior nostril. Gill membranes united and free from isthmus. Branchiostegal membrane continuous with operculum (Fig. 4A, D). Subopercular spine poorly developed (Fig. 4G).

**Table 3:** Select measurements for *Acyrtus lanthanum,* new species, *A. artius* and *A. rubiginosus*.

	*Acyrtus lanthanum*, n. sp. (n = 10)				*Acyrtus artius* (n = 10)			*Acyrtus rubiginosus* (n = 10)		
	Holotype	Range	Mean	St. Dev.	Range	Mean	St. Dev.	Range	Mean	St. Dev.
**Standard Length (SL)**										
**In % of SL**										
Head length (HL)	43.3	39.6–43.3	41.6	1.5	44.2–47.5	45.5	1.0	32.8–39.9	36.8	2.0
Body depth	16.4	15.2–18.0	16.5	0.9	14.1–18.2	15.6	1.3	13.6–16.1	14.8	0.9
Predorsal length	67.1	64.1–73.1	67.8	2.7	65.1–71.1	68.6	1.9	61.8–71.9	68.4	3.0
Preanal length	74.6	71.4–77.1	74.2	2.0	67.2–78.5	72.8	3.9	66.8–80.3	73.3	3.8
Preanus length	62.7	59.3–65.4	62.4	2.1	58.4–67	62.7	2.6	56.8–66.7	61.4	2.8
Anus to disc	7.4	4.6–8.9	6.2	1.7	8.2–15.3	11.6	2.2	12.7–14.7	14.1	0.6
Anus to anal fin	12.7	10.9–14.6	12.8	1.2	8.1–13.9	11.2	1.9	12.7–14.7	9.8	1.4
Caudal peduncle length	11.9	8.5–11.9	9.9	1.4	9.1–15.2	11.6	1.6	9.6–13.3	11.3	1.3
Caudal peduncle depth	10.4	9.7–14.1	10.7	1.4	7.7–10.5	9.5	0.9	7.0–9.6	8.3	0.7
Disc length	–	30.9–39.0	34.8	2.3	29.3–36.1	32.6	2.1	27.3–31.7	28.9	1.7
Disc width	–	31.0–38.3	35.2	2.4	29.9–34.9	32.7	1.6	22.0–30.7	27.4	2.6
**In % of HL**										
Head depth at orbit	55.2	50.5–59.6	53.9	2.6	37.7–47.6	43.5	2.9	39.6–45.9	43.1	2.2
Head width at orbit	82.7	77.9–89.0	82.4	3.1	68.8–77.9	73.7	3.5	77.6–92.0	84.7	4.7
Head width at widest point	98.3	96.6–105.6	99.9	2.8	81.4–101.1	93.6	5.4	90.4–109.4	101.5	5.0
Interorbital width	18.9	15.6–21.3	18.2	1.9	14.3–21.9	18.1	2.3	18.8–25.8	22.2	2.1
Snout length	25.8	22.5–30.0	25.8	2.5	20.7–27.0	24.6	1.9	26.0–32.3	29.8	2.1
Eye diameter	25.8	24.2–31.2	26.3	2.0	24.2–31.5	28.9	1.9	23.0–28.2	25.9	1.8

“–” indicates measurement was not taken.

**Table 4:** Select meristic characters for four species of *Acyrtus* (*A*. *lanthanum,* new species, *A. artius, A. pauciradiatus* and *A. rubiginosus*) and *Arcos nudus*.

	Dorsal-Fin rays					Anal-fin rays			
	7	8	9	10		6	7	8	9
*Acyrtus lanthanum,* n. sp.	–	3	2	–		–	2	**3**	–
*Acyrtus artius*	–	–	**4**	–		–	1	**3**	–
*Acyrtus rubiginosus*	–	–	**12**	1		–	–	**13**	1
*Acyrtus pauciradiatus*	x	x	–	–		x	x	x	–
*Arcos nudus*	–	**4**	–	–		–	**4**	–	–
	**Pectoral–fin rays**								
	**20**	**21**	**22**	**23**	**24**	**25**	**26**	**27**	**28**
*Acyrtus lanthanum,* n. sp.	–	–	–	–	1	**4**	–	–	–
*Acyrtus artius*	–	–	–	–	1	**2**	–	1	–
*Acyrtus rubiginosus*	–	–	–	–	4	**5**	2	1	–
*Acyrtus pauciradiatus*	x	x	x	–	–	–	–	–	–
*Arcos nudus*	–	–	–	1	**2**	**1**	–	–	–
	**Vertebrae**								
	**Abdominal**					**Caudal**			
	**10**		**11**	**12**		**14**	**15**	**16**	**17**
*Acyrtus lanthanum,* n. sp.	–		1	**4**		–	**4**	1	–
*Acyrtus artius*	–		1	**3**		2	2	–	–
*Acyrtus rubiginosus*	–		**13**	1		–	–	4	**10**
*Arcos nudus*	–		**4**	–		1	**3**	–	–

Data for *A. pauciradiatus* taken from Sampaio et al. [15].

**Figure 5:**
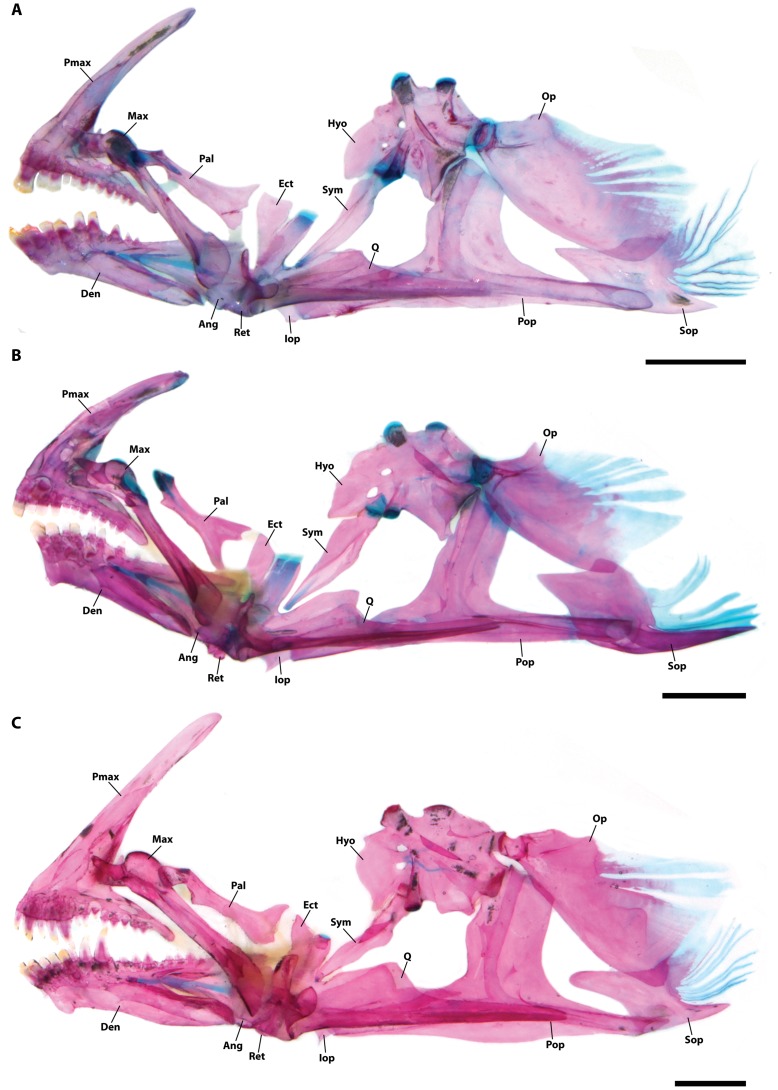
Suspensorium (right side in lateral view; images reversed) in members of *Acyrtus*. A, *Acyrtus lanthanum,* new species, UF 212697, 18.0 mm SL. B, *Acyrtus artius*, ANSP 123658, 18.0 mm SL; dorsal head of quadrate damaged. C, *Acyrtus rubiginosus*, UF 149202, 20.0 mm SL. Abbreviations: Ang, anguloarticular; Den, dentary; Ect, ectopterygoid; Hyo, hyomandibular; Iop, interopercle; Max, maxilla; Op, opercle; Pal, autopalatine; Pmax, premaxilla; Pop, preopercle; Ret, retroarticular; Sop, subopercle; Sym, symplectic; Q, quadrate. Scale bars equal to 1 mm.

Mouth terminal, posterior tip of upper jaw not reaching vertical through anterior margin of orbit when mouth closed. Upper lip fleshy, widest anteriorly, separated from snout by deep groove. Lower lip with pair of low fleshy lobes centrally. Upper jaw with 2+2 (3 specimens) or 3+4 (1) blunt to weakly trifid incisiform teeth anteriorly, followed by a single row of 6–8 small coniform teeth (Fig. 5A). Lower jaw with 4+4 (3) or 5+4 (1) blunt to weakly trifid incisiform teeth anteriorly, followed by single row of 5–6 coniform teeth along crest of dentary. Coniform teeth in upper and lower jaws decreasing in size posteriorly; 1–2 anteriormost conical teeth in lower-jaw row distinctly larger than those located more posteriorly (Fig. 5A). Pharyngeal jaws comprising patch of 8–12 small coniform teeth on pharyngobranchial toothplate 3 and a single row of 10–12 small coniform teeth along ceratobranchial 5.

Cephalic lateral-line system with 2 pores in nasal canal; 2 pores in postorbital canal; 3 pores in lachrymal canal; 3 pores in preopercular canal; and 2 pores in mandibular canal.

Dorsal-fin rays 8 (3) or 9 (2). Anal-fin rays 7(2) or 8(3). Principal caudal-fin rays 5+5, procurrent rays 5+4 (1), 5+5 (2), 6+5 (1) or 6+6 (1). Pectoral-fin rays 24 (4) or 25 (1). Pelvic-fin rays I, 4. All fin rays, excluding anteriormost dorsal- and anal-fin rays, unbranched and segmented. Anteriormost dorsal- and anal-fin rays singular, unbranched and unsegmented elements. Total number of vertebrae 27, consisting of 11+16 (1) or 12+15 (4). First dorsal-fin pterygiophore inserting between neural spines of vertebrae 11 and 12 (1), 12 and 13 (3) or 13 and 14 (1). First anal-fin pterygiophore inserting between hemal spines of vertebrae 12 and 13 (1), 13 and 14 (1) or 14 and 15 (3). Ribs 10, associated with vertebrae 3–13. Epicentrals 11, associated with vertebrae 3–14.

**Figure 6:**
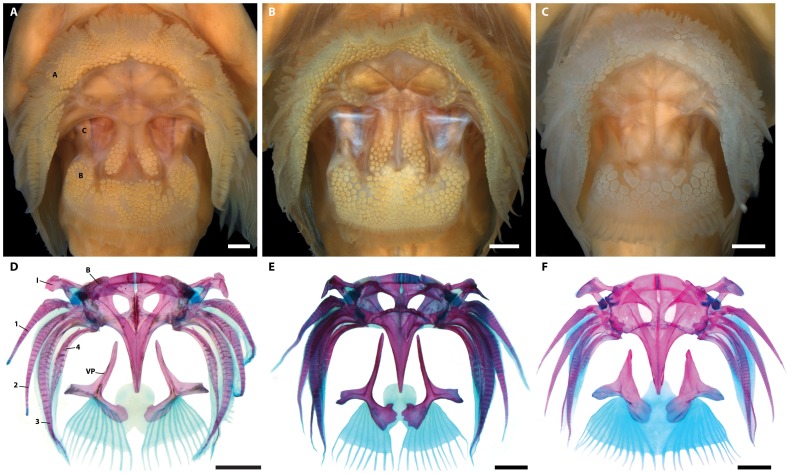
External surface (A–C) and skeletal structure (D–F) of the adhesive disc in members of *Acyrtus*. A, *Acyrtus lanthanum,* new species, ANSP 106336, 20.4 mm SL. B, *Acyrtus artius*, ANSP 94757, 24.2 mm SL. C, *Acyrtus rubiginosus*, UF 149202, 20.0 mm SL. D, *Acyrtus lanthanum,* new species, UF 212697, 18.0 mm SL; head of right pelvic-fin spine damaged. E, *Acyrtus artius*, ANSP 123658, 18.0 mm SL; anterior margin of left basipterygium damanged. F, *Acyrtus rubiginosus*, UF 149202, 20 mm SL. Letters A-C in A refer to disc regions as defined by Briggs (1955). Abbreviations in D: B, basipterygium; I, pelvic-fin spine; VP, ventral postcleithrum; 1–4, pelvic-fin rays 1–4. Scale bars equal to 500 µm (A–C) or 1 mm (D–F).

Adhesive disc large, singular (Fig. 6A); anterior and posterior margins crenulate. 7–8 transverse rows of papillae across width of disc region A. 10–11 transverse rows of papillae across width of disc region B. 3–4 longitudinal rows of papillae across width of disc region C. Ventral postcleithra trifid (Fig. 6D); anterior arm slender, equal in thickness along entire length; posteromedial and posterolateral arms expanded distally; tip of posteromedial arm rounded. Skin associated with last pelvic-fin ray attaching to base of pectoral fin opposite 4^th^ lowermost pectoral-fin ray. Tips of 15–17 uppermost pectoral-fin rays free, extending past interradial membranes. No fleshy pad on lateral surface of pectoral base. Caudal fin truncate. Dorsal-fin origin situated slightly anterior to vertical through anal-fin origin. Last dorsal- and anal-fin rays connected to body via a small membrane.

### Coloration

In preservative (Fig. 2), body and head pale yellow, without obvious markings or pigmentation. Prior to fixation (Fig. 3A), body background whitish and largely translucent. Three prominent saddles of pigment along dorsal midline, saddles mostly gold to dark red but sometimes reflecting green coloration; first saddle situated midway between occiput and dorsal-fin origin; second situated at dorsal-fin origin; third situated at caudal-fin base. Dorsal saddles extending ventrally over lateral surface of body as broad bars; anteriormost bar not extending onto ventral surface; two posterior bars connect with antimere at ventral midline, forming complete rings around body. Entire dorsal and lateral surface of trunk covered in small orange to red spots or dash-like markings, most obvious between saddles along dorsal midline and between bars on lateral surface of body; these markings absent from ventral region of trunk anterior to anal-fin origin. Dorsal, anal and caudal fins with orange to faint orange dash-like markings arranged in regular bands across fin surfaces. Base of pectoral fin lightly speckled with small dark erythrophores; remainder of pectoral fin and pelvic fins hyaline. Head densely covered with small pale to dark erythrophores, some of these aggregating into short vertical or oblique bars on lateral aspect of head. Small blue iridophores scattered across lateral and dorsal surfaces of head. Iris dark blue to black, with golden or dark red inner ring.

### Distribution and Habitat

*Acyrtus lanthanum* is known presently only from shallow coastal areas (lagoons and coral rubble zones ≤7 m) off Belize, the Bahamas, Puerto Rico (US), and Turks and Caicos Islands (Fig. 1A).

### Etymology

From the Greek λανθάνειν (lanthanein), to lie hidden, escape notice, in reference to the fact that this species has previously been confused with a close relative, *A. artius*. A noun in apposition.

### Common Name

“Orange-spotted clingfish” is in reference to the small orange to red spots or dash-like markings on the lateral surface of body that distinguish *A. lanthanum* from *A. artius*, which has larger, more oblong, and denser dashes.

### Remarks

Our DNA voucher material for *Acyrtus lanthanum* is restricted to material collected from the coast of Belize. Though morphological characters are consistent across the material of *Acyrtus lanthanum* examined from Belize, the Bahamas, Puerto Rico (US), and Turks and Caicos Islands, pending genetic analysis of samples from non-Belizean sites, we have chosen to restrict the type series of this species to material from Belize to correspond with available DNA vouchers.

### *Acyrtus artius* Briggs

*Acyrtus artius* Briggs [17]: 126, figures 37, 109.

### Material Examined

*Antigua:* ANSP 106112, 1, 8.3 mm SL, English Harbour, off Charlotte Point, 17° 0′ 0.00″ N, 61° 45′ 0.00″ W, J.C. Tyler & W.N. Eschmeyer, 21 July 1965. *Bahamas*: (non-DNA vouchers) ANSP 81299, 3, 13.3–25.7 mm SL, Green Cay (North of Rose Island), coral head ca ¼ mile North of cay, 25° 7′ 2.00″ N, 77° 11′ 18.00″ W, 0–45 ft, field number B-414, J. Böhlke et al., 21 July 1957; ANSP 94757, 3, 14.8–24.2 mm SL, Green Cay (North of Rose Island), coral head ca ¼ mile North of center of cay, 25° 7′ 6.00″ N, 77° 11′ 32.00″ W, 50 ft, field number B-513, J. Böhlke et al., 14 November 1959; ANSP 106336, 7 (1 c&s), 16.1–21.6 mm SL, Conception Island, isolated composite coral head off large bay on northwest end of island, 23° 50′ N, 75° 7′ W, 0–25 ft, field number B-589, J. Böhlke et al., 2 June 1962; ANSP 106338, 9, 8.6–20.5 mm SL, Hogsty Reef, isolated coral head off westernmost tip of northwestern cay, 21° 40′ N, 73° 50′ W, field number B-580, J. Böhlke et al., 29 May 1962; ANSP 143248. 1, 19.0 mm SL, Great Bahama Bank, Nassau vicinity, north of eastern half of Green Cay, 25° 7′ 0.00″ N, 77° 11′ 0.00″ W, J.E.Böhlke et al., 27 August 1969. *Belize*: (DNA vouchers) USNM 403479, DNA number BZE 8258, 16.5 mm SL, South end of South Cut, 16° 45′ 43.00″ N, 88° 4′ 27.00″ W, 12–14 m, field number CB08–21, C. Baldwin et al., 22 May 2008; USNM 403481, DNA number BZE 7814, 19.0 mm SL, Carrie Bow Cay, 16° 48′ 8.00″ N, 88° 4′ 54.00″ W, 8–11 m, field number CB07-83, C. Baldwin et al., 1 October 2007; USNM 403483, DNA number BLZ 7815, 13.0 mm SL, Carrie Bow Cay, 16° 48′ 8.00″ N, 88° 4′ 54.00″ W, 8–11 m, field number CB07-83, C. Baldwin et al., 1 October 2007; USNM 403491, DNA number BLZ 8043, 17.0 mm SL, Curlew outer ridge, 16° 47′ 24.00″ N, 88° 4′ 41.00″ W, 25 ft., field number CB08-02, C. Baldwin et al., 15 May 2008; USNM 403492, DNA number BLZ 8042, 17.0 mm SL, Curlew outer ridge, 16° 47′ 24.00″ N, 88° 4′ 41.00″ W, 25 ft., field number CB08-02, C. Baldwin et al., 15 May 2008; USNM 403494, DNA number BLZ 8109, 8.0 mm SL, Glovers, southwest Cay East wall, 16° 42′ 36.00″ N, 87° 51′ 5.00″ W, 15–24 m, field number CB08-10, C. Baldwin et al., 18 May 2008; USNM 403498, DNA number BZE 8257, 19.0 mm SL, South end of South Cut, 16° 45′ 43.00″ N, 88° 4′ 27.00″ W, 12–14 m, field number CB08-21, C. Baldwin et al., 22 May 2008; USNM 404119, DNA number BLZ10119, 7.5 mm SL, South end of South Cut, 16° 45′ 45.00″ N, 88° 4′ 30.00″ W, 15–20 m, field number CB10-14, C. Baldwin & M. Fagan-Halloran, 13 November 2010; USNM 404162, DNA number BLZ 10162, 20.0 mm SL, Carrie Bow Cay, South of South Cut, 16° 45′ 46.00″ N, 88° 4′ 26.00″ W, 10–17 m, field number CB10-17, C. Baldwin & M. Fagan-Halloran, 14 November 2010; USNM 404163, DNA number BLZ 10163, 9.5 mm SL, Carrie Bow Cay, South of South Cut, 16° 45′ 46.00″ N, 88° 4′ 26.00″ W, 10–17 m, field number CB10-17, C. Baldwin & M. Fagan-Halloran, 14 November 2010; USNM 404205, DNA number BLZ 10205, 19.0 mm SL, Carrie Bow Cay, near South of South Cut, lat long, 12–17 m, field number CB10-22, C. Baldwin & M. Fagan-Halloran, 15 November 2010. (non-DNA vouchers) FMNH 83936, 5, 6.7–16.5 mm SL, Stann Creek, Curlew Cay, R.K. Johnson et al., 13 March 1980; FMNH 83939, 3, 10.4–15.6 mm SL, Corozal, Ambergris Cay, first cut in barrier reef North of San Pedro, ca. 1.5mi North of San Pedro, D.W. Greenfield et al., 10 June 1980; FMNH 83940, 3, 10.5–18.11 mm SL, Corozal, Ambergris Cay, cut in reef ca. 2.5mi North of San Pedro, R.K. Johnson et al., 11 July 1980; FMNH 83942, 4, 9.9–15.0 mm SL, Corozal, Ambergris Cay, first cut to South of San Pedro, R.K. Johnson et al., 12 July 1980; FMNH 84321, 3, 12.6–16.7 mm SL, Glover’s Reef, between Long Cay and Middle Cay, R.K. Johnson & G.S. Glodek, 13 June 1978; FMNH 84323, 9.6–16.8 mm SL, Glover’s Reef, Long Cay, just above top of dropoff at South end, R.K. Johnson et al., 14 June 1978; FMNH 84324, 4, 14.9–20.4 mm SL, Glover’s Reef, South of Long Cay, near top of dropoff, D.W. Greenfield et al., 15 June 1978; FMNH 84329, 3, 14.9–16.8 mm SL, Glover’s Reef, near top of dropoff off NE Cay, D.W. Greenfield et al., 28 June 1979; FMNH 84331, 7, 7.7–16.6 mm SL, Glover’s Reef, near top of dropoff at Southwest Cay, D. W. Greenfield et al., 30 June 1979. *Cayman Islands:* ANSP 123692, 2, 17.4–18.8 mm SL, Grand Cayman Island, Paradise Rocks, offshore from North side of Georgetown, C.R.Gilbert & J.C.Tyler, 22 October 1964. *Curaçao*: CAS-SU 23254, 1, holotype (only photograph and x-ray examined), 18.2 mm SL, Caracas Bay, C.J. Van Der Horst, 3 May 1920. *Haiti*: (non-DNA vouchers) ANSP 123658, 10 (2 c&s), 12.0–20.9 mm SL, Gulf of Gonave, St. Marc Channel, off Mount Rouis, 2 miles southeast of Mount Rouis town, 18° 55′ N, 72° 39′ W, 0–7 ft, field number TFD-7, J. Tyler, H. Feddern & T. Devany, 15 September 1967. *Trinidad and Tobago*: (DNA vouchers) USNM 403505, DNA number TOB 9193, 1, 14.5 mm SL, Buccoo Reef, 11° 11′ N, 60° 50′ W, 15–18 m, C. Baldwin, D. Smith, L. Weigt, 17 Mar 2009. *Turks and Caicos Islands*: USNM 403493, 16.0 mm SL, Fish Bowl, South Caicos, 21° 29′ 6.00″ N, 71° 30′ 30.00″ W, 15–20 m, field number TCI09-08, C. Baldwin et al., 9 October 2009.

### Diagnosis

A member of the genus *Acyrtus* distinguished from all congeners except *A. pauciradiatus* by the presence of a deep pocket between the branchiostegal membrane and the operculum; a large opaque patch of skin associated with the medial face of the subopercle (caused by a dense aggregation of large, tightly packed clavate cells in the epidermis); an elongate, ventrally grooved subopercular spine that contributes to over half of the total length of the subopercle; and two widely separated clusters of papillae along the anterolateral margin of disc region C, each comprising 7–12 small, closely associated papillae. It is distinguished from *A. pauciradiatus* by its higher number of pectoral-fin rays (24–27 vs. 20–22) and fresh color pattern, notably the presence of several mostly dark red saddles on the trunk (vs. uniform pale reddish pink trunk). *Acyrtus artius* is further distinguished from *A. lanthanum* usually by having a longer head (head length 44–47% SL vs. 40–43), a narrower head (head width at orbit 68–78% HL vs. 78–89%) and a shallower head (head depth at orbit 38–48% HL vs. 50–60% HL); by the shape of the ventral postcleithrum (tip of posteromedial arm scalloped vs. rounded); and by fresh color pattern (dash-shaped orange to red markings on trunk large, oblong and dense vs. smaller, more rounded and less dense; dashes extending ventrally to region anterior to anal-fin origin vs. terminating dorsal to that region; bar or blotch of pigment between the saddle of pigment at the base of the caudal fin and the one beneath the anterior portion of the dorsal fin vs. absence of bar or blotch of pigment here; and iris uniformly red or with red blotches vs. dark blue/black with gold to red inner ring). From *A. rubiginosus, A. artius* is distinguished by its larger adhesive disc (disc length 29–36% SL vs. 27–32% SL), the absence of large coniform teeth anterolaterally in upper jaw (vs. presence), fewer teeth in the lower jaw (3–4 small coniform teeth posterior to larger, anterolaterally placed coniform teeth vs. 7–8), a single row of pharyngeal teeth associated with ceratobranchial 5 (vs. two rows), the presence of paired clusters of papillae posteriorly in disc region C (vs. absence), by having small round papillae along the anterior edge of disc region B (vs. large, irregular shaped papillae), by the shape of the ventral postcleithra (anterior arm slender and uniform in diameter along entire length vs. anterior arm thicker proximally, decreasing in diameter distally), and by fresh color pattern, most notably in having a mostly white background body color (vs. purple).

### Description

**Figure 7:**
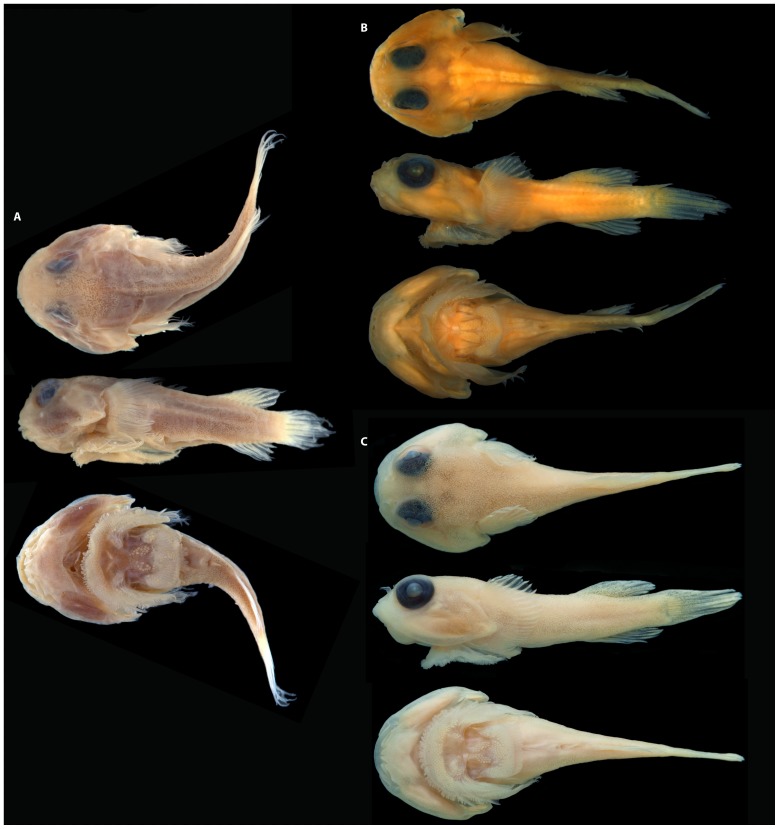
*Acyrtus artius*. A, holotype, CAS-SU 23254, 18.4 mm SL, Curacao. B, FMNH 84329, 15.4 mm SL, Belize. C, ANSP 106336, 20.4 mm SL, Bahamas. Photographs in A by Jon Fong (CAS).

General body shape as in figures 3B and 7. Morphometric data are listed in Table 3 and selected counts in Table 4. Head large, slightly dorsoventrally compressed. Body moderately dorsoventrally compressed anteriorly; becoming increasing laterally compressed posteriorly. Body deepest midway between head and dorsal-fin origin. Eye large, positioned in upper half of head; center of eye closer to tip of snout than to posterior margin of operculum. Snout short. Anterior nostril tubular, with small, often bifurcated, cirri extending from posterior margin. Posterior nostril surrounded by low fleshy rim; situated close to base of anterior nostril. Gill membranes united and free from isthmus. Deep pocket between branchiostegal membrane and operculum (Fig. 4B, E), lined laterally by a large opaque patch of skin (caused by a dense aggregation of large, tightly packed clavate cells in epidermis) associated with medial face of subopercle. Subopercular spine well developed; grooved ventrally (Fig. 4I).

Mouth terminal, posterior tip of upper jaw reaching vertical through anterior margin of orbit when mouth closed. Upper lip fleshy, widest anteriorly, separated from snout by deep groove. Lower lip with pair of low fleshy lobes centrally. Upper jaw with 4+4 (3 specimens) or 4+5 (1) blunt to weakly trifid incisiform teeth anteriorly, followed by a single row of 6–8 small coniform teeth (Fig. 5B). Lower jaw with 4+4 (3) or 4+5 (1) blunt to weakly trifid incisiform teeth anteriorly, followed by single row of 5–6 coniform teeth. Coniform teeth in upper and lower jaws decreasing in size posteriorly; 2–3 anteriormost conical teeth in lower-jaw row distinctly larger than those located more posteriorly (Fig. 5B). Pharyngeal jaws comprising patch of 4–8 small coniform teeth on pharyngobranchial toothplate 3 and a single row of 3–6 small coniform teeth along ceratobranchial 5.

Cephalic lateral-line system with 2 pores in nasal canal; 2 pores in postorbital canal; 3 pores in lachrymal canal; 3 pores in preopercular canal; and 2 pores in mandibular canal.

Dorsal-fin rays 9. Anal-fin rays 7(1) or 8(3). Principal caudal-fin rays 5+5, procurrent rays 5+5 (3) or 6+5 (1). Pectoral-fin rays 24(1), 25(1) or 27(2). Pelvic-fin rays I, 4. All fin rays, excluding anteriormost dorsal- and anal-fin rays, unbranched and segmented. Anteriormost dorsal- and anal-fin rays singular, unbranched and unsegmented elements. Total number of vertebrae 26(3)–27(1), consisting of 11+15(1), 12+14(2) or 12+15(1). First dorsal-fin pterygiophore inserting between neural spines of vertebrae 11 and 12 (2) or 12 and 13 (2). First anal-fin pterygiophore inserting between hemal spines of vertebrae 13 and 14 (2) or 14 and 15 (2). Ribs 8 (3) or 9 (1), associated with vertebrae 3–10 (3) or 3–11 (1). Epicentrals 11 (2) or 12 (2), associated with vertebrae 3–13 (2) or 3–14 (2).

Adhesive disc large, singular (Fig. 6B); anterior and posterior margins crenulate. 7–8 transverse rows of papillae across width of disc region A. 10–11 transverse rows of papillae across width of disc region B. 3–4 longitudinal rows of papillae across width of disc region C. Anterolateral margin of disc region C with two widely separated clusters of 7–10 papillae. Ventral postcleithra trifid (Fig. 6E); anterior arm slender, equal in thickness along entire length; posteromedial and posterolateral arms expanded distally; tip of posteromedial arm scalloped. Skin associated with last pelvic-fin ray attaching to base of pectoral fin opposite 4^th^ lowermost pectoral-fin ray. Tips of 15–17 uppermost pectoral-fin rays free, extending past interradial membranes. No fleshy pad on lateral surface of pectoral base. Caudal fin truncate. Dorsal-fin origin situated slightly anterior to vertical through anal-fin origin. Last dorsal- and anal-fin rays connected to body via a small membrane.

### Coloration

As described for *Acyrtus lanthanum* in preservative, with the following differences prior to fixation (Fig. 3B): dash-like, orange-red markings on dorsal and lateral surfaces of body and median fins larger, more oblong, and more densely covering areas between saddles; several dashes present on ventral portion of trunk anterior to origin of anal fin; bar or blotch of dark orange to red pigment between saddle of pigment at base of caudal fin and one beneath anterior portion of dorsal fin; and iris dark red or with dark orange/red blotches, grading to light red or pale yellow inner ring.

### Distribution and Habitat

*Acyrtus artius* occurs at depths of 8–20 m on spur-and-groove structure and on walls of outer ridges throughout the Bahamas and Caribbean region (Fig. 1A). To date, we have examined material of *Acyrtus artius* from Antigua, Bahamas, Belize, Cayman Islands, Curaçao, Haiti, Turks and Caicos Islands, and Trinidad and Tobago (Tobago) (Fig. 1A). The holotype (CAS-SU 23254) represents the only available record of *Acyrtus artius* from Curaçao. Recent efforts by one of us (CB) to collect specimens of *Acyrtus* at the type locality in Curaçao (Caracas Bay) and nearby waters resulted only in specimens of *Acyrtus rubiginosus*. In addition, we were unable to locate the single paratype of *Acyrtus artius* (USNM 78158) reported to be from St. Thomas by Briggs [17], which (if belonging to this species) represents the only record of *Acyrtus artius* from the Virgin Islands.

### Remarks

The holotype of *Acyrtus artius* (CAS-SU 23254; Fig. 7A) is in poor condition, and the papillae of the adhesive disc are badly eroded. In this specimen, the branchiostegal membrane is laterally concave, creating a deep pocket between it and the operculum. The skin lining the lateral wall of this pocket (i.e., the skin lining the medial face of the subopercle) is opaque and appears granular. The subopercle also bears a well-developed, ventrally grooved, spine-like process, which has been misidentified as the preopercular spine [17]. We have observed this combination of features in *Acyrtus artius* from throughout the Caribbean and Bahamas (Fig. 4B, E. I), but it is not present in *Acyrtus lanthanum* (Fig. 4A, D, G).

Several specimens of *Acyrtus artius* that we have examined are in excellent condition, and the papillae covering the surface of the adhesive disc are intact or nearly so (e.g., Fig. 6B). In addition to having the paired clusters of papillae in the posteromedial region of disc region C (for which the species is named [17]), these well-preserved individuals also exhibit two widely separated clusters of papillae along the anterolateral margin of disc region C, a region generally devoid of papillae in members of the Gobiesocidae [17]. Though these latter papillae are not present in all individuals of *Acyrtus artius* that we have examined (including the holotype; Fig. 7A), we interpret this to be the result of damage or rough handling after collection rather than a polymorphic characteristic of the species. Notably, we also have identified paired clusters of papillae along the anterolateral margin of disc region C in the holotype of *Acyrtus pauciradiatus* (examined from photographs only) but not in *Acyrtus lanthanum* (Fig. 6A) or *Acyrtus rubiginosus* (Fig. 6C), which may be indicative of a close relationship between *Acyrtus artius* and *Acyrtus pauciradiatus*.

Böhlke and Chaplin [21] suspected that *Acyrtus artius* would be conspecific with *Arcos macrophthalmus,* with the former representing juveniles of the latter. *Arcos macrophthalmus* is now recognized as *Arcos nudus* [40]. Though superficially similar, *Acyrtus artius* is easily distinguished from *Arcos nudus* by differences in adhesive disc papillae, including the presence of two widely separated clusters of papillae along the anterolateral margin of disc region C (vs. absent in *Arcos nudus*) and by having fewer papillae in the paired clusters posteriorly in disc region C (24–30 papillae per cluster, arranged in 3–4 rows in *Acyrtus artius* vs. 68–90 papillae per cluster, arranged in 8–10 rows in *Arcos nudus*). *Acyrtus artius* can be further distinguished from *Arcos nudus* based on differences in upper jaw dentition. In *Acyrtus artius*, the premaxilla bears only incisiform teeth anteriorly that are flanked laterally by small coniform teeth (Fig. 5B). Contrary to Briggs [17] incisiform teeth are absent from the upper jaw in the material of *Arcos nudus* that we have examined, with the premaxilla instead bearing large caniniform teeth anteriorly that are flanked laterally by smaller coniform teeth. *Acyrtus artius* can be distinguished with confidence from *Arcos nudus* by differences in body length. The largest individual of *Arcos nudus* that we have examined is 81 mm SL (ANSP 118638), over three times the length of the largest individual of *Acyrtus artius* examined (26 mm SL, ANSP 81299). Finally, *Acyrtus artius* and *Arcos nudus* have very different color patterns, with the latter having a mostly green-yellow pigment pattern with red/orange bars restricted to the caudal fin (Fig. 3D; see also http://www.fishbase.org/photos/PicturesSummary.php?ID=16663&what=species).

## Discussion

In their treatment of the clingfishes of Belize and Honduras, Johnson & Greenfield [22] provided an overview of *Acyrtus artius*, including information on habitat at numerous collection locations and a summary of external measurements obtained from 61 individuals. Johnson and Greenfield [22] noted (pg. 38) a strong correlation between head length and depth of capture in their Belizean material of *Acyrtus artius*, with specimens from deeper water having “proportionally larger heads” than specimens collected from shallower water. They [22] speculated that this relationship between head length and collection depth could reflect “differential grow rates at different depths” but did not investigate this phenomenon further.

Based on our detailed morphological and molecular investigation of *Acyrtus* from Belize and throughout the western Atlantic, it is now clear that Johnson and Greenfield’s treatment of *Acyrtus artius* was derived from specimens belonging to two different species of *Acyrtus*, including the real *Acyrtus artius* (from deeper water) and a very similar looking species (from shallower water), which we have described herein as *Acyrtus lanthanum*. Like Johnson & Greenfield, we also had originally considered all *Acyrtus* with paired patches of papillae in disc region C to represent *Acyrtus artius* (as have the majority of other investigators working with western Atlantic clingfishes [17–19, 21, 41–42). It was only through the examination of DNA sequences collected from specimens originally identified as *Acyrtus artius*, which revealed the existence of two highly divergent lineages, that we were encouraged to take a closer look at *Acyrtus artius*, resulting in the discovery of the new species, *Acyrtus lanthanum*.

**Figure 8:**
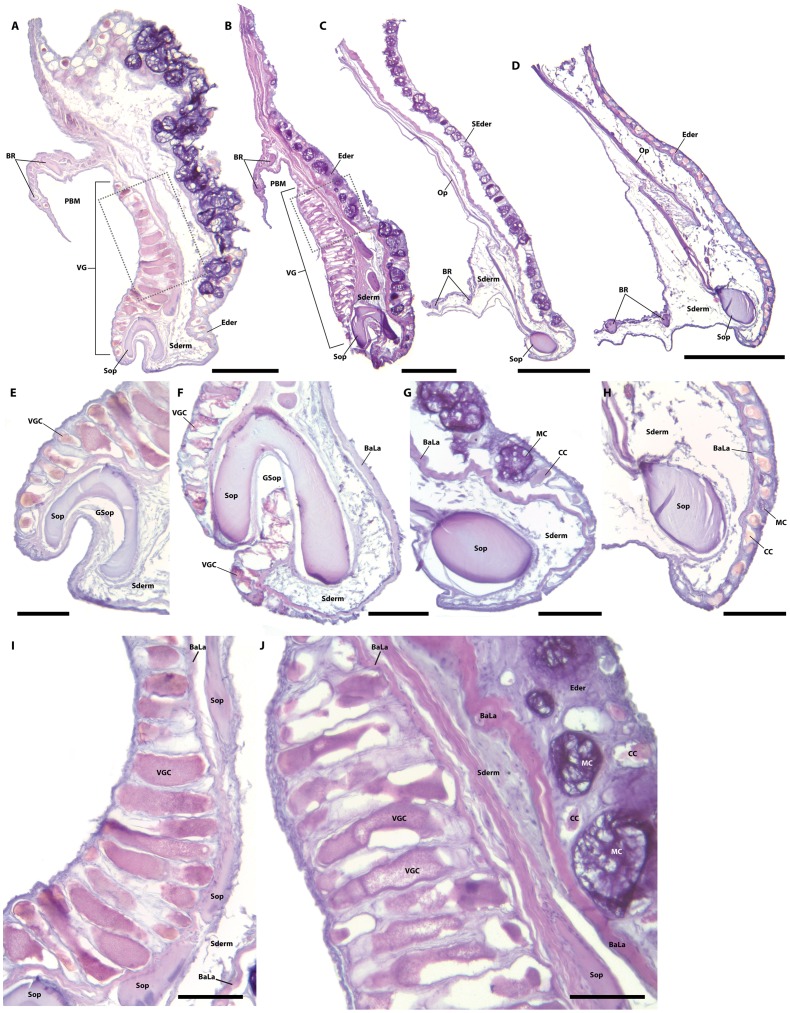
Sections through the subopercular region in species of *Acyrtus* and *Arcos*. A, *Acyrtus artis,* ANSP 106336, 19.0 mm SL. B, *Arcos nudus,* ANSP 94773, 37.1 mm SL. C, *Acyrtus lanthanum,* new species, ANSP 81310, 19.5 mm SL. D, *Acyrtus rubiginosus* ANSP 106128, 18.0 mm SL. E, close up of subopercular spine; same specimen as in A. F, close up of subopercular spine; same specimen as in B. G, close up of subopercular spine; same specimen as in C. H, close up of subopercular spine; same specimen as in D. I, Close up of box in A, showing cells of venom gland. J, Close up of box in B, showing cells of venom gland. Abbreviations: BaLa, basal lamina of epidermis; BR, branchiostegal ray; CC, club cell; Ederm, epidermis; GSop, groove in subopercle; MC, mucus cell; Op, opercle; PBM, pocket in branchiostegal membrane; SEderm, sloughed epidermis; Sderm, subdermis; Sop, subopercle; VG, venom gland; VGC, venom gland cell. Scale bars equal to 500 µm (A–D) or 100 µm (E–J).

Without a doubt, the most notable differences between *Acyrtus lanthanum* and *Acyrtus artius* relate to modifications of the subopercular region in the latter, including a well-developed and ventrally grooved subopercular spine (Fig. 4I), and a deep pocket in the branchiostegal membrane, between the 6^th^ branchiostegal ray and the subopercle, that is lined laterally by an opaque patch of skin (Fig. 4B, E). In *Acyrtus lanthanum* the subopercular spine is poorly developed and is circular in cross-section (Fig. 4G, 8G), the branchiostegal membrane is continuous with the operculum, and there is no obvious differentiation of the skin between the 6^th^ branchiostegal ray and the subopercle (Fig. 4D). A similar arrangement is present in *Acyrtus rubiginosus*, except that the subopercular spine is more robust (Fig. 4H, 8D). Though we have not had the opportunity to examine specimens of *Acyrtus pauciradiatus* (known to date only from the Fernando de Noronha Archipelago of the coast of North East Brazil [19]), examination of photographs taken of the holotype (MZUSP 84516) reveal that, like *Acyrtus artius,* this species also exhibits a well-developed subopercular spine associated with a deep pocket in the adjacent branchiostegal membrane that is lined by an opaque patch of skin. Unexpectedly, our investigation of *Arcos nudus* has revealed this combination of features also to be present in this species.

Histological investigation of the opaque patch of skin lining the lateral wall of the pocket in the branchiostegal membrane of *Acyrtus artius* and *Arcos nudus* reveals its opacity to be caused by a dense aggregation of large, pillar-shaped secretory cells in the epidermis; the contents of which stain intensely eosinophilic (Fig. 8A, B). These cells are over ten times larger than other epidermal secretory cells (mucus or club cells [43]) in adjacent regions of the epidermis and occupy most of the epidermal space where they are found (from the basal lamina to the thin layer of squamous cells lining the surface of the epidermis; Fig. 8I, J). Spatially, this cluster of large cells lines the entire lateral wall of the pocket in the branchiostegal membrane and is closely associated with the inner (medial) edge of the subopercular spine. A smaller cluster of large secretory cells, separate from the larger cluster lining the lateral wall of the pocket in the branchiostegal membrane, is also present inside the groove of the subopercular spine in *Acyrtus artius*. This smaller cluster of large secretory cells is restricted entirely to the anteriormost portion of the groove and is replaced posteriorly by a very thin epidermis that is devoid of secretory cells, combined with a thin layer of loose connective tissue derived from the subdermis (Fig. 8E). In *Arcos nudus*, large secretory cells are also present inside the groove in the subopercular spine (Fig. 8F) but extend almost the entire length of the groove, being absent only at the posteriormost tip of the spine.

Though we have not investigated the function of the large secretory cells in the epidermis of *Acyrtus artius* and *Arcos nudus*, they are very similar in appearance to the toxin-producing or clavate cells that are present in the venom glands of teleost fishes [43]–[45]. Based on this similarity, we identify these large secretory cells as venom-producing cells, and tentatively identify the well-developed and ventrally grooved subopercular spine as the delivery mechanism for this venom. Though we have not been able to examine specimens of *Acyrtus pauciradiatus*, we predict (based on the presence of an opaque patch of skin in close association with the subopercular spine in the holotype; MZUSP 84516) that this species exhibits a venom apparatus similar to that present in *Acyrtus artius* and *Arcos nudus*. In the strict consensus tree resulting from the parsimony analysis of the COI dataset (Fig. 1B), *Acyrtus artius* is recovered as the sister group to *Acyrtus lanthanum* (with moderate bootstrap support), and together those species form part of a trichotomy with *Acyrtus rubiginosus* and *Arcos nudus* (this clade lacks bootstrap support). Though largely unresolved, the relationships within this *Arcos*/*Acyrtus* clade are somewhat perplexing given that *Acyrtus artius* and *Arcos nudus*, characterized by highly unusual modifications of the subopercle and adjacent integument, are not recovered as a monophyletic group. Additional data for all western Atlantic *Acyrtus* and eastern Pacific *Arcos* (*Arcos decoris*, *Arcos erythrops*, *Arcos poecilophthalmos* and *Arcos rhodospilus* [46]) are needed, but possibly the putative venom apparatus diagnoses a clade of clingfishes that includes members currently classified in both *Acyrtus* and *Arcos.*

Given the detailed morphological studies of clingfishes by Briggs [17] and others in the nearly 60 and 260 years, respectively, since the original descriptions of *Acyrtus artius* [17] and *Arcos nudus* [47], it is remarkable that the unusual configuration of the subopercle and associated glandular tissue in these two species have not previously been reported. Though venom glands are widespread amongst acanthomorph teleosts [44], [48]–[49], putative venom glands have not been reported previously for the Gobiesocidae, nor have venom glands been reported previously in association with the subopercle for any other group of teleost fishes [49]. Our study represents only the most recent of a series of anatomical studies, spanning the last fifty years, which have resulted in the discovery of novel groups of venomous or potentially venomous fishes [45], [49]–[53]. Given that toxic compounds are known from the skin of gobiesocids [54], further investigation of the secretory cells in the venom glands of western Atlantic clingfishes is warranted.

Lastly, our study has also revealed relatively high intraspecific variation in the COI gene for *Acyrtus rubiginosus* (3.5%) and *Acyrtus artius* (1.3%), with the largest differences occurring between specimens from Belize and those from Tobago and Bahamas (*A. rubiginosus*) or between Belize and Tobago (*A. artius*) (see Figure 1B and Table 1). Additional material from the Bahamas and eastern Caribbean is needed to determine if there are more cryptic species of *Acyrtus* clingfishes within the western Atlantic.

## Supporting Information

Table S1 **DNA number, museum voucher number, Genbank number and GenSeq designation** [34] **for COI sequences utilized herein.** For museum collection abbreviations see Sabaj Pérez [25].
(DOCX)

Information S1 **List of museum material examined.**
(DOCX)
