# Supplementary material for: Cryptic Diversity and Venom Glands in Western Atlantic Clingfishes of the Genus Acyrtus (Teleostei: Gobiesocidae)
Source: PLoS One. 2014 May 13;9(5):e97664. doi: 10.1371/journal.pone.0097664 (PMC4019652; doi:10.1371/journal.pone.0097664)
Supplement: Table S1 — DNA number, museum voucher number, Genbank number and GenSeq designation [34] for COI sequences utilized herein. For museum collection abbreviations see Sabaj Pérez [25]. (DOCX) [file pone.0097664.s001.docx]

**Table S1.** DNA number, museum voucher number, Genbank number and GenSeq designation [34] for COI sequences utilized herein. For museum collection abbreviations see Sabaj Pérez [25].

| **Species** | **DNA number** | **Museum Voucher** | **Genbank Number** | **GenSeq Designation** |
| --- | --- | --- | --- | --- |
| *Acyrtus lanthanum* n. sp. | BLZ 7231 | USNM 403477 | KJ616416 | genseq-2 COI |
|  | BLZ 7282 | USNM 403490 | KJ616417 | genseq-2 COI |
|  | BLZ 7283 | USNM 403480 | KJ616418 | genseq-2 COI |
|  | BLZ 7284 | (Photo voucher only) | KJ616419 | genseq-5 COI |
|  | BLZ 8136 | USNM 403499 | KJ616420 | genseq-2 COI |
|  | BLZ 8185 | USNM 403478 | KJ616421 | genseq-2 COI |
|  | BLZ 8215 | USNM 403189 | KJ616422 | genseq-2 COI |
|  | BLZ 8222 | USNM 403497 | KJ616423 | genseq-2 COI |
|  | BLZ 10132 | USNM 404132 | KJ616424 | genseq-2 COI |
|  | BLZ 10133 | USNM 404133 | KJ616425 | genseq-2 COI |
|  | BLZ 10171 | USNM 404171 | KJ616426 | genseq-2 COI |
|  | BLZ 10173 | USNM 404173 | KJ616427 | genseq-2 COI |
| *Acyrtus artius* | BLZ 5329 | USNM 420346 | KJ616428 | genseq-4 COI |
|  | BLZ 7814 | USNM 403481 | KJ616429 | genseq-4 COI |
|  | BLZ 7815 | USNM 403483 | KJ616430 | genseq-4 COI |
|  | BLZ 8042 | USNM 403492 | KJ616431 | genseq-4 COI |
|  | BLZ 8043 | USNM 403491 | KJ616432 | genseq-4 COI |
|  | BLZ 8109 | USNM 403494 | KJ616433 | genseq-4 COI |
|  | BLZ 10119 | USNM 404119 | KJ616434 | genseq-4 COI |
|  | BLZ 10162 | USNM 404162 | KJ616435 | genseq-4 COI |
|  | BLZ 10163 | USNM 404163 | KJ616436 | genseq-4 COI |
|  | BLZ 10205 | USNM 404205 | KJ616437 | genseq-4 COI |
|  | BZE 8257 | USNM 403498 | KJ616438 | genseq-4 COI |
|  | BZE 8258 | USNM 403479 | KJ616439 | genseq-4 COI |
|  | TOB 9193 | USNM 403505 | KJ616440 | genseq-4 COI |
| *Acyrtus rubiginosus* | BAH 8195 | USNM 403486 | KJ616441 | genseq-4 COI |
|  | BAH 8196 | USNM 403495 | KJ616442 | genseq-4 COI |
|  | BAH 8197 | USNM 403485 | KJ616443 | genseq-4 COI |
|  | BLZ 7331 | USNM 403503 | KJ616444 | genseq-4 COI |
|  | BLZ 10174 | USNM 404174 | KJ616445 | genseq-4 COI |
|  | BLZ 10176 | USNM 404176 | KJ616446 | genseq-4 COI |
|  | TOB 9344 | USNM 403484 | KJ616447 | genseq-4 COI |
| *Arcos nudus* | ELU 1003 | USNM 403507 | KJ616448 | genseq-4 COI |
| *Acyrtops beryllinus* | KWC-AB1 | TCWC 15701.01 | KJ616449 | genseq-4 COI |
| *Gobiesox strumosus* | KWC-GS1 | TCWC 16453.01 | KJ616450 | genseq-4 COI |
| *Gobiesox maeandricus* | EJH08-8-1 | VIMS 12257 | KJ616451 | genseq-4 COI |
| *Rimicola muscarum* | KWC-RM1 | SIO 03-40 | KJ616452 | genseq-4 COI |
| *Sicyases sanguineus* | KWC-SS1 | ANSP 191454 | KJ616453 | genseq-4 COI |
| *Tomicodon briggsi* | BLZ 8135 | USNM 421892 | KJ616454 | genseq-4 COI |
| *Tomicodon reitzae* | TOB 9076 | USNM 421704 | KJ616455 | genseq-4 COI |
| *Apletodon dentatus* | KWC-ApD1 | BMNH uncat. | KJ616456 | genseq-4 COI |
| *Lepadogaster purpurea* | LR GO101 | NMBE uncat. | KJ616457 | genseq-4 COI |
| *Parvicrepis parvipinnis* | KWC-PP2 | AM I.44125-041 | KJ616458 | genseq-4 COI |
|  |  |  |  |  |
