# Supplementary material for: Cryptic Diversity and Venom Glands in Western Atlantic Clingfishes of the Genus Acyrtus (Teleostei: Gobiesocidae)
Source: PLoS One. 2014 May 13;9(5):e97664. doi: 10.1371/journal.pone.0097664 (PMC4019652; doi:10.1371/journal.pone.0097664)
Supplement: Information S1 — List of museum material examined. (DOCX) [file pone.0097664.s002.docx]

**Supporting Information 1 –** List of museum material examined.

*Acyrtus rubiginosus*

*Bahamas:* (DNA vouchers) USNM 403485, DNA number BAH 8197, 1, 19.0 mm SL, Great Harbour Cay, Sugar Beach/cave, C. Baldwin et al., 18 June 2008; USNM 403486, DNA number BAH 8195, 1, 23.0 mm SL, Great Harbour Cay, Sugar Beach/cave, C. Baldwin et al., 18 June 2008; USNM 403495, DNA number BAH 8196, 1, 20.0 mm SL, Great Harbour Cay, Sugar Beach/cave, C. Baldwin et al., 18 June 2008; (non DNA vouchers) ANSP 115200, 1, 25.5 mm SL, Hog Island, New Providence, 20 March 1951; ANSP 81333, 58, 7.0-23.7 mm SL, Exuma Cays; small rock-sided, sandy-beach baylet on south east side of Warderick Wells Cay, 24° 23' 5.00" N, 76° 36' 55.00" W, 18 July 1957; UF 107840, 28, 6.8-26.8 mm SL, Andros Islands, E.C. Jones, 19 June 1951. *Belize*: (DNA vouchers) USNM 403503, DNA number BZE 7331, 1, 16.0mm SL, Carrie Bow Cay, L. Weigt, 21 January 2007; USNM 404131, DNA number BLZ10131, 1, 13.5 mm SL, Carrie Bow Cay, south side, C.Castillo & D.Griswold, 13 November 2010; USNM 404174, DNA number BLZ 10174, 1, 10.5 mm SL, Carrie Bow Cay, south side, C.Castillo & D.Griswold, 14 November 2010; USNM 404175, DNA number BLZ10175, 1, 9.0 mm SL, Carrie Bow Cay, south side, C.Castillo & D.Griswold, 14 November 2010; USNM 404176, DNA number BLZ10176, 1, 6.5 mm SL, Carrie Bow Cay, south side, C.Castillo & D.Griswold, 14 November 2010; USNM 404186, DNA number BLZ10186, 1, 18.5 mm SL, Carrie Bow Cay, 16° 46' 15" N, 88° 07' 00" W, C.Castillo & D.Griswold, 14 November 2010; USNM 404187, DNA number BLZ10187, 1, 15.5 mm SL, Carrie Bow Cay, 16° 46' 15" N, 88° 07' 00" W, C.Castillo & D.Griswold, 14 November 2010; (non DNA vouchers) FMNH 83943, 4, 7.4-18.3 mm SL, Corozal, Ambergris Cay, south of cut in reef 2.5mi North of San Pedro, R.K. Johnson et al., 11 July 1980. *Cuba*: MCZ 12923, 1, syntype of *Sicyases rubiginosus*, 17.9 mm SL, Matanzas; MCZ 12925, 6, syntypes of *Sicyases carneus,* 12.9-21.9 mm SL, Matanzas; MCZ 34153, 1, holotype of *Sicyases yumurina*, 22.8 mm SL, Matanzas; MCZ 34154, 2, paratypes of *Sicyases yumurina*, 20.6-20.7 mm SL, Matanzas. *Dominican Republic*: FMNH 61695, 2, 16.7-16.8 mm SL, Beata Bay, near False Cape beach, 17° 45' 0.00" N, 71° 32' 0.00" W, D.S. Erdman, 11 November 1953. *Grenada*: ANSP 106123, 8, 10.8-24.7 mm SL, Martins Bay (south of St. George Harbor), south end of bay, 12° 2' 0.00" N, 61° 46' 0.00" W, 21 June 1965. *Haiti*: FMNH 61694, 2, 8.3-15.4 mm SL, Bigie Bay, Bigie Point at noth end of bay, 18° 25' 0.00" N, 74° 29' 0.00" W, D.S. Erdman, 28 October 1953. *Honduras*: FMNH 84337, 27, 11.4-24.4 mm SL, Cayos de Cochinos Menor, R.K. Johnson et al., 18 May 1975; FMNH 84338, 3, 13.2-21.0 mm SL, Cayos de Cochinos Menor, D.W. Greenfield et al., 19 May 1975; FMNH 84340, 14, 11.9-21.0 mm SL, Cayos de Cochinos Grande, R.R. Miller et al., 21 May 1975. *Puerto Rico (US):* ANSP 144522, 12, 11.4-26.2 mm SL, Isla Desecheo, rocky shore in small bay on south west side, 6 March 1965. *St. Barthelemy*: ANSP 106127, 44, 7.4-26.2 mm SL, Isle Syndare, Port de Gustavia; W edge of smaller of two islets of Syndares; 0-1.5 m; from boulders and seagrass, 17° 55' 0.00" N, 62° 54' 0.00" W, 13 July 1965; FMNH 62069, 5, 15.5-24.7 mm SL, Isla Desecheo, D.S. Erdman & V. Biaggi, 29 June 1952. *St. Lucia:* ANSP 106128, 37 (3 c&s), 7.4-21.1 mm SL, Port Castries, at Vigie Point, 14° 2' 0.00" N, 61° 0' 0.00" W, 3 July 1965. *Trinidad and Tobago*: (DNA vouchers) USNM 403484, DNA number TOB 9344, 1, 38.0 mm SL, Arnos Vale Beach, 11° 13' 36.00" N, 60° 45' 50.00" W, C. Baldwin et al., 19 March 2009; (non DNA vouchers) ANSP 98485, 2, 22.0 mm SL, Tobago, east side of Goat Island, 27 April 1962. *Turks and Caicos Islands*: (DNA vouchers) USNM 403490, DNA number TCI 9603, 1, 18.0 mm SL, South Caicos, Dove Cay, L.Weigt & C.Castillo, 12 October 2009; USNM 403500, DNA number TCI 9604, 1, 13.0 mm SL, South Caicos, Dove Cay, L.Weigt & C.Castillo, 12 October 2009. *Virgin Islands (UK):* ANSP 94764, 12, 14.0-21.4 mm SL, Little Camanoe Island, west end of South Beach, 21 July 1959. *Virgin Islands (US)*: UF 149202, 62 (7 examined, 2 c&s), 10.7-22.2 mm SL, St. Croix, Northwest shore of Buck Island REEF National Monument, 17 47 24 N, 64 37 29 W, 23 July 2001.

*Acyrtus pauciradiatus:* MZUSP 84516, 1 (photograph only), holotype 16.3 mm SL, Brazil, off State of Pernambuco, Fernando de Noronha Archipelago, Rata Island, in Ressurreta Bay, at a depth of 12m, L.F. Mendes, 12 December 1997.

*Arcos nudus*

*Bahamas:* (DNA vouchers) USNM 403507, DNA number ELU 1003, 1, 49.7 mm SL, West side of Whale Point, L. Johnson, 26 September 2009; (non DNA vouchers) ANSP 81309, 1, 24.5 mm SL, Hog Island, east of Cabbage Beach, north shore, 25° 5' 31.00" N, 77° 18' 48.00" W, C.C.G. Chaplin, 5 March 1953; ANSP 116148, 1, 67.4 mm SL, Exumas, east side of small cay south of Compass Cay, H.A. Feddern et al., 24 August 1963. *Puerto Rico (US)*: ANSP 115602, 10, 12.7-74.7 mm SL, 1.3 miles south south west of Playa de Guayanes, Municipio de Yabucoa, N.R. Foster et al., 12 July 1969; ANSP 118638, 5, 20.7-81.0 mm SL, 1.3 miles south south west of Playa de Guayanes, Municipio de Yabucoa, N.R. Foster et al., 20 January 1971; ANSP 129882, 3, 9.5-59.5 mm SL, Puerto Yabucoa, 1.3 miles south south west of Playa de Guayanes, Municipio de Yabucoa, J.J. Loos, 24 July 1973; FMNH 61696, 2, 43.1-51.1 mm SL, Isla Desecheo, D.S. Erdman & V. Biaggi, 29 June 1952. *Trinidad and Tobago:* ANSP 142945, 37, 12.7-56.4 mm SL, Tobago, shore at Mt. Irvine, J. Randall & H. Randall, 3 May 1964. *Turks and Caicos Islands:* USNM 403488, 1, 18.0 mm SL, South Caicos, Dove Cay, 21° 29' 6.00" N, 71° 31' 53.00" W, L. Weigt and C. Castillo, 12 October 2009. *Virgin Islands (UK)*: ANSP 94773, 6, 37.1-55.0 mm SL, Guana Island, Money Point, south end of island facing narrow strait between Guana and Tortola island, D.M. Barringer & A.N. Barringer, 22 July 1959.
